# Practical Recommendations for Anticoagulation in Patients With Atrial Fibrillation

**DOI:** 10.1111/eci.70224

**Published:** 2026-05-18

**Authors:** Nicolas Johner, Baris Gencer

**Affiliations:** ^1^ Cardiology Division Geneva University Hospitals Geneva Switzerland; ^2^ Hôpital Cardiologique du Haut‐Lévêque, CHU de Bordeaux Pessac France; ^3^ Cardiology Division Lausanne University Hospital Lausanne Switzerland; ^4^ Institute of Primary Healthcare (BIHAM) University of Bern Bern Switzerland

**Keywords:** anticoagulation, atrial fibrillation, bleeding, direct oral anticoagulant, stroke, thromboembolism, vitamin K antagonist

## Abstract

**Background:**

The lifetime risk of stroke among non‐anticoagulated patients with atrial fibrillation (AF) approximates 1 in 3. Oral anticoagulation (OAC) reduces stroke and systemic embolism by at least two thirds and mortality by one fourth.

**Methods:**

The present narrative review summarizes current evidence and practical recommendations on OAC in AF and highlights recent advances and remaining gaps in knowledge.

**Results:**

The threshold for net clinical benefit of OAC is met when stroke risk exceeds 1%–2% per year, typically corresponding to ≥ 1 traditional stroke risk factor (CHA_2_DS_2_‐VA ≥ 1), with increasing risk per additional risk factor. OAC also appears beneficial in device‐detected (subclinical) AF, possibly at higher CHA_2_DS_2_‐VA scores. Direct oral anticoagulants (DOACs) reduce intracranial haemorrhage compared to vitamin K antagonists (VKAs), and meta‐analyses of randomized trials showed further reduction in stroke and mortality with DOACs, including in the elderly and chronic kidney disease. VKAs are preferred in patients with mechanical heart valves, mitral stenosis, antiphospholipid syndrome and Child‐Turcotte‐Pugh C cirrhosis. Modifiable bleeding risk factors should be assessed periodically and mitigated. These include anti‐inflammatory drugs, antiplatelet therapy, drug interactions, excessive alcohol consumption, uncontrolled hypertension, diabetes, risk factors for gastrointestinal bleeding, major organ dysfunction and frailty. There are very few contraindications to OAC and most are relative or temporary conditions. Follow‐up involves reassessing adherence, thrombotic and bleeding risk, co‐medication and dosing. Integrated patient‐centred AF management additionally involves risk factor management, symptom control and dynamic reassessment. Lifelong OAC is currently recommended, but recent data suggested that discontinuing OAC after successful catheter ablation of AF and/or left atrial appendage closure could be safe. OAC in specific settings is discussed, including cardioversion, catheter ablation, surgery, post‐operative AF, elderly patients, pregnancy and bleeding.

**Conclusion:**

OAC is the cornerstone of thromboembolism prevention in AF, but knowledge gaps remain, including on risk stratification, device‐detected AF, trigger‐induced AF, cardioversion, OAC resumption after major bleeding, and potential safety of OAC discontinuation after successful catheter ablation or left atrial appendage occlusion.

## Graphical abstract

Oral anticoagulation (OAC) reduces stroke risk by at least two thirds and mortality by one fourth. The threshold for net clinical benefit of OAC is met when stroke risk exceeds 1%–2% per year. Direct oral anticoagulants (DOACs) are preferred to vitamin K antagonists (VKAs) in most patients, including in the elderly and chronic kidney disease. Modifiable bleeding risk factors should be periodically assessed and mitigated. Most contraindications to OAC are relative or temporary. Periodic follow‐up with systematic reassessment is recommended.

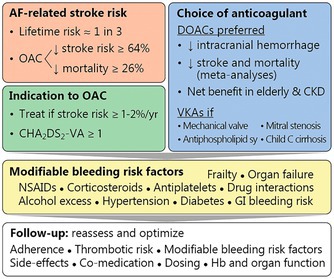

## 1 Introduction

With a lifetime risk exceeding 1 in 4 persons [1, 2], atrial fibrillation (AF) is the most common cardiac arrhythmia and a major cause of morbidity, hospitalization and death. AF is associated with a twofold increase in mortality [3] and a fivefold increase in stroke risk [4]. Among AF patients, the lifetime risk of stroke without anticoagulation is approximately 1 in 3 [2, 5] and AF is estimated to be responsible for 15%–25% of all strokes [4, 6, 7, 8]. Compared to other etiologies (e.g., atherosclerotic), AF‐related strokes are associated with greater permanent disability, higher recurrence risk and greater mortality [4, 9]. The mainstay of thromboembolic risk mitigation, and the most established prognosis‐modifying therapy in AF, is long‐term oral anticoagulation (OAC) [10, 11]. In older studies, placebo‐controlled randomized trials have shown that vitamin K antagonists (VKA) reduce stroke risk by 64% and mortality by 26% [12]. Observational data also showed a 29% reduction in dementia [13], possibly explained by the prevention of otherwise silent brain infarcts [14]. While the benefit of OAC in AF is established, real‐world management has been suboptimal [15] in terms of prescription and time in therapeutic range, and areas of uncertainty remain regarding practical issues and specific clinical settings. Recent expert recommendations highlight the importance of a systematic, patient‐centred, integrated approach to AF management to optimize best‐practice implementation. In the 2024 ESC guidelines [11], these principles are outlined in the structured AF‐CARE framework, which includes four pillars: [C] comorbidity and risk factor management, [A] avoid stroke and thromboembolism, [R] reduce symptoms by rate and rhythm control and [E] evaluation and dynamic reassessment. The present narrative review summarizes the current evidence and practical recommendations on anticoagulation in patients with AF. Recent advances, remaining gaps in knowledge and future perspectives are discussed. Key practical takeaways and areas of uncertainty are specifically highlighted. Focus is therefore primarily directed at the [A] pillar of integrated AF care, while also providing targeted insight into other aspects of AF management from the perspective of anticoagulation, including comorbidity management [C], symptom mitigation by rhythm control [R] and follow‐up [E]. Institutional review board approval was not required as no experiments were conducted.

## 2 Thromboembolic Risk Assessment and Indications to Anticoagulation

### 2.1 Clinical AF

The risk of stroke and systemic embolism may be categorized as low (< 1%/year), intermediate (1%–2%/year) or high (≥ 2%/year) [16]. OAC is indicated in all AF patients, except those at low thromboembolic risk. The net clinical benefit of OAC is well established in patients at high risk [17, 18, 19, 20, 21, 22, 23]. Patients at intermediate risk generally also benefit from OAC [17, 20], but inaccuracy in thromboembolic risk estimation and heterogeneity in this population has led to weaker recommendations for OAC in these patients (class IIa recommendation) [11, 16]. A Markov state transition decision model [24] based on data from warfarin trials and the RE‐LY trial [17] found that the tipping point for net benefit of OAC was 1.7% annual stroke risk for warfarin and 0.9% for dabigatran. Several risk scores have been developed to quantify thromboembolic risk in clinical practice. The CHA_2_DS_2_‐VASc score [25] is considered the most validated and has been used in prospective trials to validate OAC efficacy. Also, cardiovascular trials testing direct oral anticoagulants (DOACs) systematically included AF patients with a CHA_2_DS_2_‐VASc ≥ 2. Female sex, however, is a risk modifier rather than an independent risk factor, and is inadequate to identify patients at low or intermediate risk (females with a CHA_2_DS_2_‐VASc score ≤ 2 have similar risk to men with a score ≤ 1), prompting the recommendation to use the CHA_2_DS_2_‐VA score instead to guide clinical decisions [11]. Recently, the CHA_2_DS_2_‐VA score was in fact shown to have marginally superior discriminative ability compared to the CHA_2_DS_2_‐VASc in modern cohorts in which sex differences have decreased compared to historical cohorts [26, 27]. The absolute thromboembolic risk associated with any given CHA_2_DS_2_‐VA(Sc) score level varies substantially between cohorts [28]. Nevertheless, for practical purposes, CHA_2_DS_2_‐VA scores of 0, 1 and ≥ 2, are generally considered to identify patients at low (< 1%/year), intermediate (1%–2%/year) and high risk (≥ 2%/year), respectively [16]. Notable exceptions include patients with hypertrophic cardiomyopathy [29] and cardiac amyloidosis [30], who are almost universally at high risk and should be prescribed OAC regardless of CHA_2_DS_2_‐VA level. Likewise, AF patients with significant mitral stenosis are generally believed to be at higher risk compared to controls with identical CHA_2_DS_2_‐VA score, prompting recommendations for OAC regardless of CHA_2_DS_2_‐VA in this population [31], even though high‐quality evidence is scarce [32]. When there is doubt about the indication to OAC (e.g., low‐intermediate estimated risk), using other scores (e.g., GARFIELD‐AF [33], ATRIA [34]) that include additional risk factors not included in the CHA_2_DS_2_‐VA (e.g., renal disease), and taking into account AF type and burden (persistent AF and greater burden increase stroke risk [35]) may help guide therapeutic decisions [16].

Of note, the diagnosis of AF is defined, by consensus, as a 12‐lead ECG showing AF (10 s) or > 30 s of AF on a single‐lead or continuous ECG tracing. ECG‐based wearable devices and other non‐invasive ECG‐based methods (e.g., smart watches, handheld devices) are therefore appropriate for AF diagnosis, provided that AF diagnosis is confirmed by a physician. In contrast, non‐ECG‐based methods (e.g., photoplethysmography) are not diagnostic of AF (but may be indicative) and require confirmation with an ECG‐based method [11].

### 2.2 Device‐Detected AF

Device‐detected AF or subclinical AF, is defined as asymptomatic AF detected by a cardiac implantable electronic device, including atrial high‐rate episodes (AHRE) of confirmed arrhythmic nature. Indications to anticoagulation in patients with device‐detected AF are not clearly established. Stroke risk is dependent on the duration and burden of device‐detected AF episodes/AHRE [36]. Episodes shorter than 5–6 min and AF burden not exceeding 5–6 min per 24 h are considered clinically insignificant and are not associated with longer episodes or thromboembolic risk [37, 38]. Episodes longer than 24 h have been associated with stroke risk approaching that of clinical AF [39] and are often managed similarly to clinical AF [40]. Device‐detected AF of intermediate duration (6 min to < 24 h) is associated with an approximately twofold increase in stroke risk, that is, 50% lower compared to clinical AF [36, 38, 41]. In this population, observational studies and post hoc analyses of randomized trials have shown that stroke rates exceed the threshold for net benefit of OAC at a CHA_2_DS_2_‐VASc score of approximately 4–5 [42, 43] (as opposed to 1–2 for clinical AF). Two randomized trials evaluated the benefit of OAC in device‐detected AF patients with a CHA_2_DS_2_‐VASc ≥ 2 (NOAH trial comparing edoxaban versus placebo or aspirin [depending on established indications for aspirin]) [44] or ≥ 3 (ARTESiA trial comparing apixaban versus aspirin) [45]. A meta‐analysis of the two trials [46] found results to be consistent (*I* ^2^ for heterogeneity = 0%), with a reduction in ischemic stroke in the OAC group (relative risk [RR], 0.68; 95% CI, 0.50–0.92). Conversely, OAC increased major bleeding (RR, 1.62; 95% CI, 1.05–2.50; *I* ^2^ = 61%). In absolute terms, OAC yielded three fewer ischemic strokes per 1000 patient‐years at the cost of seven more major bleeding events per 1000 patient‐years. It may therefore be reasonable to prescribe OAC in selected patients with device‐detected AF lasting 6 min to < 24 h based on shared decision making, particularly in patients with higher thromboembolic risk (e.g., CHA_2_DS_2_‐VASc score ≥ 4–5, higher device‐detected AF burden) and/or vascular disease, for whom the net benefit of OAC appears greater [42, 47, 48]. Of note, there was indeed greater net benefit of OAC in patients with a CHA_2_DS_2_‐VASc score > 4 in the ARTESiA trial [42], but no significant difference in the NOAH trial [48]. The 2024 ESC guidelines provide a class IIb recommendation for OAC in device‐detected AF with elevated thromboembolic risk [11]. In addition, patients with device‐detected AF are at high risk of disease progression [49] and should therefore be closely followed up for the development of higher AF burden and clinical AF [11].

### 2.3 Post‐Operative and Trigger‐Induced AF

New‐onset AF in the setting of an acute precipitating factor is associated with a lower risk of AF recurrence compared to new‐onset AF without acute trigger, but still carries approximately 40% risk of AF recurrence at 5 years [50]. Comparing types of triggers, acute medical illness, such as sepsis, myocardial infarction, or critical care, seems associated with the highest risk of long‐term AF recurrence, followed by non‐cardiac surgery, while cardiac surgery is associated with the lowest risk of recurrence [50].

Observational studies suggest that post‐operative AF after non‐cardiac surgery is associated with a similar increase in long‐term thromboembolic risk compared to the general AF population [51]. Regarding post‐operative AF following cardiac surgery, there is conflicting data on long‐term thromboembolic risk, and continuous monitoring has shown that AF burden approaches 0% beyond post‐operative Day 30 [52, 53]. Still, meta‐analyses of observational studies suggest that long‐term OAC could reduce thromboembolic events and all‐cause mortality in post‐operative AF after cardiac surgery [54, 55]. Ongoing randomized trials, including PACES (NCT04045665) and ASPIRE‐AF (NCT03968393), will likely clarify this area of uncertainty. In the meantime, it may be reasonable to introduce OAC as soon as deemed safe from a surgical standpoint and to consider long‐term OAC (especially after non‐cardiac surgery) or to reassess the indication to long‐term OAC at 60 days depending on rhythm status (especially after cardiac surgery) [11, 16].

New‐onset AF in the setting of acute medical illness has been associated with similar thromboembolic risk as AF without precipitant [56] and long‐term anticoagulation after resolution of the acute illness may be reasonable (class IIa recommendation) [11]. However, the introduction of OAC during the acute phase of sepsis is of unclear net benefit [57].

Patients with hyperthyroidism and AF are at increased thromboembolic risk, and OAC is recommended based on standard thromboembolic risk evaluation [16, 56]. New‐onset AF in the setting of hyperthyroidism most commonly resolves within 3 weeks of normalization of thyroid function [58]; as a result, it may be reasonable to discontinue OAC when thyroid function is normalized and if durable sinus rhythm can be maintained [16].

## 3 Addressing Modifiable Bleeding Risk Factors

Bleeding is the most common adverse event resulting from anticoagulation. All patients who are anticoagulated should undergo periodic assessment of bleeding risk so that modifiable risk factors are identified and, if possible, mitigated [59]. Table 1 summarizes common modifiable and partially modifiable bleeding risk factors along with corresponding bleeding reduction strategies. Clinical risk scores that include some of these conditions have been developed to help assess bleeding risk (e.g., HAS‐BLED [60], HEMORR_2_HAGES [61], ATRIA [62]). Importantly, high bleeding risk as estimated by such scores does not represent a contraindication to anticoagulation and OACs should not be withheld in such circumstances. To avoid under‐use of anticoagulation, the 2024 ESC clinical practice guidelines and the 2023 ACC/AHA/ACCP/HRS guideline recommend against the use of bleeding risk scores to decide on initiating or withdrawing OAC (class III recommendation) [11, 16].

**TABLE 1:** Modifiable and partially modifiable bleeding risk factors for patients on OAC.

Risk factor	Bleeding reduction strategy
NSAIDs	Discontinue NSAID, chose alternative if available
Corticosteroid drugs	Minimal effective dose, discontinue as early as possible
Antiplatelet therapy	Discontinue as early as possible after acute coronary syndrome/last angioplasty as per guideline‐recommended treatment duration
Labile INR	Switch to DOAC if possible Optimize time in therapeutic range (verify adherence, dietary intake of vitamin K, optimize education, review interactions with drugs and co‐morbid conditions)
Drug–drug interactions	Review co‐medication periodically and avoid significant interactions if possible
Excessive alcohol consumption	Education, counselling, refer to specialized management if necessary
Repetitive falls	Integrated care, refer to specialized management if necessary
Frailty	Integrated care, refer to specialized management if necessary
High‐risk activities	Avoid activities at high risk of trauma
Thrombocytopenia	Management of underlying cause, periodic re‐assessment
Unexplained anaemia	Identify and treat underlying cause
Uncontrolled hypertension	Optimize antihypertensive therapy, aim for guideline‐recommended targets
High gastrointestinal bleeding risk	Proton pump inhibitor for patients with risk factors: – History of gastrointestinal bleeding – History of peptic ulcer – Gastro‐oesophageal reflux disease – *Helicobacter pylori* infection – Cirrhosis – Dyspepsia – Advanced age – Excessive alcohol consumption – Antiplatelet therapy – NSAID – Corticosteroid drugs
Diabetes	Optimize glycemic control and risk factor management
Heart failure	Guideline‐directed medical therapy Euvolemia
Chronic kidney disease	Closer follow‐up of renal function Evidence‐based OAC dose adjustment Specific management of underlying disease
Liver disease/cirrhosis	Avoid rivaroxaban in patients with Child‐Turcotte‐Pugh B cirrhosis All DOACs contraindicated in Child‐Turcotte‐Pugh C cirrhosis (use VKAs) Proton pump inhibitor Screen for high‐risk oesophageal varices Close follow‐up of organ function, clinical status, co‐medication, alcohol intake Discuss OAC discontinuation (with multidisciplinary team and patient) in case of recent major bleeding, severe thrombocytopenia, active coagulopathy, high‐risk varices not suitable for intervention Specific management of underlying disease
Cancer	Closer follow‐up of organ function, clinical status Specific management of underlying disease

Abbreviations: DOAC, direct oral anticoagulant; INR, international normalized ratio; NSAID, non‐steroidal anti‐inflammatory drug; OAC, oral anticoagulation; VKA, vitamin K antagonist.

## 4 Contraindications to Anticoagulation

There are very few absolute contraindications to anticoagulation and most are relative or temporary. Possible contraindications to OAC and factors to consider in such settings are summarized in Table 2. When there is uncertainty about the risk–benefit ratio of OAC, decisions regarding the initiation, withdrawal or resumption of OAC and its timing are best made within a multidisciplinary team (including cardiologists and specialists involved in the management of the condition at risk of bleeding) and should take into account the severity, cause, management and reversibility of the condition that may contraindicate anticoagulation [11]. Notably, most patients identified as having relative contraindications to OAC and/or high bleeding risk still derive net benefit from OAC [5, 63, 64]. When OAC is considered durably contraindicated, left atrial appendage (LAA) closure is an option to mitigate thromboembolic risk (class IIb recommendation in ESC guidelines) [11]. Randomized trials have shown non‐inferior protection against stroke and systemic embolism compared to OAC in selected AF patients [65]. Interestingly, catheter ablation of AF might also reduce long‐term stroke risk [66] and recent randomized trials have shown that OAC could be safely discontinued after successful catheter ablation (no documented atrial arrhythmia recurrence for ≥ 1 year) in selected patients [67, 68]. However, catheter ablation is not currently part of the established antithrombotic armamentarium and further studies are needed to clarify the optimal antithrombotic strategy following successful catheter ablation.

**TABLE 2:** Possible contraindications to OAC and settings with unfavourable risk–benefit ratio.

Possible contraindication	Determining factors
Intracranial or spinal tumours	Location and type of tumour Treatment
History of intracranial haemorrhage	Underlying cause (e.g., amyloid angiopathy and spontaneous subdural hematoma are associated with very high rebleeding risk) Time since bleeding event Reversibility, treatment of underlying cause
Severe thrombocytopenia < 50 G/L	Platelet count and trend Platelet function Underlying cause
Severe bleeding diathesis	Severity and nature of hypocoagulable state Reversibility, treatment (e.g., clotting factor prophylaxis combined with OAC might be indicated in selected patients, while others may be considered naturally anticoagulated)
Recent or active major bleeding	Site (critical sites include intracranial, intraspinal, intraocular, intra‐articular, pericardial, airway, hemothorax, retroperitoneal, intra‐abdominal, intramuscular with compartment syndrome) Severity (uncontrolled or life‐threatening) Time since bleeding has stopped Reversibility of underlying cause (e.g., source of bleeding identified and treated)
Severe liver disease with additional high‐risk features	Recent major bleeding, active coagulopathy, severe thrombocytopenia, high‐risk varices not suitable for intervention In the absence of these high‐risk features, most patients appear to derive net clinical benefit of OAC (observational data)
End‐of‐life care	Life expectancy < 6–12 months (competitive risk of non‐AF mortality)
Invasive procedures	Category of procedure‐related bleeding risk (minimal/low/high) according to incidence of bleeding, ease of controlling bleeding and risk of adverse outcome if bleeding occurs Renal function (for dabigatran, adjust duration of pre‐operative OAC accordingly)
Delivery	Planned delivery is recommended for anticoagulated patients VKAs, LMWH and UFH should be discontinued 2 weeks, 24 h and 4–6 h before delivery, respectively

Abbreviations: INR, international normalized ratio; LMWH, low molecular weight heparin; NSAID, non‐steroidal anti‐inflammatory drug; OAC, oral anticoagulation; UFH, unfractionated heparin; VKA, vitamin K antagonist.

## 5 Choice of Anticoagulant

DOACs (apixaban, dabigatran, edoxaban and rivaroxaban) have all shown at least non‐inferior efficacy compared to VKAs (e.g., warfarin, acenocoumarol, phenprocoumon) for the prevention of AF‐related systemic embolism as well as a significantly lower risk of intracranial bleeding [17, 19, 20, 21]. A meta‐analysis of DOAC pivotal trials including a total of 71,683 patients found that, compared to warfarin, DOACs further reduced stroke risk by 19%, all‐cause mortality by 10% and intracranial bleeding by 52%, while other major bleeding events did not significantly differ [23]. DOACs additionally reduced non‐cerebral systemic embolism by 29% compared to VKAs [69]. Importantly, the favourable risk–benefit profile of DOACs compared to VKAs seems preserved or even superior in patients at higher bleeding risk such as elderly and frail patients [70, 71, 72]. Given this outcome data, the ease of use, and predictable pharmacologic profile, the default strategy should be to prefer DOACs over VKAs in all eligible patients [11, 16]. Exceptions include patients with significant mitral valve stenosis [73], mechanical heart valves [74, 75] and antiphospholipid syndrome [76], for whom randomized trials have shown VKAs to be superior to DOACs. In addition, all DOACs are contraindicated in patients with Child‐Turcotte‐Pugh C cirrhosis (rivaroxaban should also not be used in Child‐Turcotte‐Pugh B cirrhosis [77]), and VKAs should be used in these patients if eligible (see also Table 1) [78].

## 6 Dosing, Monitoring and Adjustment to Renal Function

The efficacy and safety of DOACs were established with standard full‐dose DOAC therapy. Dose reduction is appropriate for patients fulfilling specific criteria as per manufacturer recommendations (Table 3). Inappropriate (nonevidence‐based) dose reduction in patients who do not meet these criteria is frequent in clinical practice and is associated with lower efficacy with no benefit in safety [85, 86], and should therefore be avoided.

**TABLE 3:** Evidence‐based and recommended doses of DOACs and VKAs according to renal function and patient characteristics [11, 16, 79, 80, 81].

	Creatinine clearance (mL/min, Cockcroft‐Gault)				
	> 95	51–95	30–50	15–29	< 15 or dialysis
Apixaban	5 mg twice daily	5 mg twice daily	5 mg twice daily	2.5 mg twice daily^a^	2.5 mg twice daily^a^
	2.5 mg twice daily if ≥ 2 of the following: serum creatinine ≥ 133 μmol/L (≥ 1.5 mg/dL), age ≥ 80 years, body weight ≤ 60 kg				
Dabigatran	150 mg twice daily	150 mg twice daily	150 mg twice daily	75 mg twice daily^b^	Not recommended
	110 mg twice daily if age ≥ 80 years or concomitant verapamil; also consider if high risk of bleeding (e.g., age 75–80 years, creatinine clearance 30–50 mL/min, gastroesophageal reflux…)				
Edoxaban	60 mg once daily^c^	60 mg once daily	30 mg once daily	30 mg once daily	Not recommended
	30 mg once daily if body weight ≤ 60 kg, concomitant potent P‐gp inhibitor				
Rivaroxaban	20 mg once daily	20 mg once daily	15 mg once daily	15 mg once daily	15 mg once daily
VKAs	INR: 2.0–3.0	INR: 2.0–3.0	INR: 2.0–3.0	INR: 2.0–3.0	INR: 2.0–3.0
				Maintenance dose typically 20% lower	
DOACs versus VKAs	DOACs preferred	DOACs preferred	DOACs preferred	Equipoise	Equipoise

*Note:* Green cells indicate established efficacy and safety, yellow indicates cautionary use, red indicates area of significant uncertainty, grey indicates not recommended use.

Abbreviations: DOAC, direct oral anticoagulant; FDA, Food and Drug Administration; USA, United States of America; VKA, vitamin K antagonist.

^a^ The 2023 ACC/AHA/ACCP/HRS Guideline recommends 5 or 2.5 mg twice daily with the same criteria for dose reduction as in patients with GFR ≥ 30 mL/min [16].

^b^ Only approved in the USA, based on pharmacokinetic simulations.

^c^ The FDA issued a warning about possible reduced efficacy of edoxaban in patients with creatinine clearance > 95 mL/min based on subgroup analysis from ENGAGE AF [82]. Subsequent studies did not reproduce these results and some authors suggested the initial subgroup findings might have been fortuitous [83, 84].

VKAs should be titrated to a target international normalized ratio (INR; patient prothrombin time divided by control prothrombin time) of 2.0–3.0 [11]. For patients with a mechanical heart valve, a higher INR target may be recommended depending on valve type, position and additional pro‐thrombotic factors [31]. Dose regimens and frequency of INR monitoring depend on specific VKAs, patient characteristics and clinical settings. Briefly, baseline INR should be measured before VKA initiation, and a lower initial dose should be used in case of coagulopathy. INR is typically monitored daily upon initiation (starting after the 2nd or 3rd VKA dose) for patients at high thromboembolic risk and/or in hospital settings. The frequency of INR monitoring can then be progressively decreased in a stepwise fashion (e.g., daily, then once every 3 days, then weekly, then fortnightly, then monthly) each time two consecutive INR results are within therapeutic range. For patients on maintenance therapy, INR is typically monitored monthly. Unpredictable fluctuations in INR values may result from VKA interaction with drugs, dietary intake of vitamin K and comorbid conditions, as well as adherence issues. When the INR is outside of therapeutic range, and if a dose modification is deemed appropriate, adjustment should be based on the cumulative dose that led to the last INR value (e.g., weekly dose for warfarin, but time frame varies with specific VKAs). Time in therapeutic range (TTR), defined as the proportion of time spent with an INR within target range, should be maximized to avoid preventable thromboembolism and bleeding [87]. A TTR > 70% is generally considered appropriate [11] but may be difficult to achieve in a substantial proportion of patients. For example, in the pivotal trials of DOACs versus VKAs, mean TTRs were < 70% in over half of participating centres [88].

Regarding patients with chronic kidney disease (CKD), VKAs have historically been considered preferable to DOACs given their hepatic metabolism. However, patients with CKD on VKA therapy exhibit lower TTRs, leading to increased risk of stroke and bleeding [89, 90]. Patients with severe CKD still require approximately 20% lower maintenance doses of VKAs [79]. Moreover, VKA therapy in CKD may be associated with increased vascular calcifications through the inhibition of vitamin K‐dependent Matrix Gla Protein (an inhibitor of vascular mineralization) [91, 92] and, rarely, calciphylaxis [93]. Finally, anticoagulant‐related nephropathy, believed to be due to glomerular haemorrhage and renal tubular obstruction as a result of supratherapeutic anticoagulation, may occur with any OAC, but VKAs have been associated with a greater risk of renal function decline compared to DOACs [94].

DOACs all exhibit partial renal elimination (approximately 27% for apixaban, 80% for dabigatran, 50% for edoxaban and 35% for rivaroxaban) [79]. Nevertheless, contemporary evidence including meta‐analyses of randomized trials consistently showed at least non‐inferior, and probably superior, efficacy and safety of DOACs compared to VKAs in patients with CKD stages 1 to 3 (glomerular filtration rate [GFR] ≥ 30 mL/min/1.73 m^2^) [95, 96, 97]. Data is scarcer for CKD stages 4 (GFR: 15–29) and 5 (GFR < 15 mL/min/1.73 m^2^) and patients undergoing dialysis [98]. In a meta‐analysis [99] of 25 nonrandomized studies and 6 randomized trials comparing DOACs to VKAs in patients with Stage 4 or 5 CKD, DOACs were associated with a lower risk of major bleeding and at least non‐inferior efficacy for the prevention of thromboembolic events. Data was more abundant and consistent for apixaban and rivaroxaban. Notably, the risk–benefit profile and net benefit of OAC itself, compared to no OAC, is not well established in this population [79, 80]. Therefore, current guidelines strongly recommend OAC, with DOACs in preference to VKAs, in patients with CKD Stages 1–3 (class I recommendation), and provide weaker recommendations for OAC in Stage 4 (class IIa recommendation) and Stage 5 CKD/dialysis (class IIb recommendation), without preference for DOACs or VKAs [16]. Evidence‐based doses of DOACs for AF in CKD are summarized in Table 3. Of note, renal function should be estimated using the Cockcroft‐Gault formula as in pivotal trials.

## 7 Follow‐Up of Patients on Oral Anticoagulants

AF patients on OACs should undergo regular and pre‐specified follow‐up. Integrated chronic care within a nurse‐led AF clinic helps coordinate follow‐up, improves adherence to guidelines and was shown to improve outcomes [100]. Drug adherence is paramount to treatment efficacy and may be as low as 25%–40% for OAC depending on settings and definitions [80, 101, 102]. Adherence should be assessed at each visit and optimized by providing education on AF and the need for OAC, the modalities of intake (dosing regimen, timing, intake with food for rivaroxaban, how to deal with a lapse in dosing), the importance of a strict intake schedule, providing information on adherence aids if necessary (e.g., pill organizers, smartphone applications), and involving family members when appropriate (e.g., frail older patients) [80]. Side‐effects should be acknowledged, information about bleeding should be provided and patients should be instructed not to skip or discontinue OAC without consulting their physician. Patient resources are available online and provide education in multiple languages (e.g., EHRA's, www.afibmatters.org). At each follow‐up visit, thromboembolic risk should be re‐assessed, which includes enquiring about intercurrent thromboembolic events. Routine blood sampling should include at least yearly assessment of haemoglobin, renal and hepatic function [80]. Co‐medication should be systematically reviewed for possible drug–drug interactions and medication that increases bleeding risk. Modifiable bleeding risk factors (Table 1) should be re‐assessed and mitigated whenever possible. Side effects and bleeding events should be carefully reviewed to identify possible precipitating factors, preventable causes and rare contraindications to OAC (Table 2). The impact on quality of life and adherence should be addressed and a change in molecule may be considered. The adequacy of the dosing regimen should be re‐evaluated based on established criteria (Table 3). Finally, the AF‐CARE framework, defined in ESC guidelines, underlines the importance of managing all other AF risk factors and associated conditions, especially obesity and alcohol consumption [11].

## 8 Management of Bleeding on Anticoagulant Therapy

Not only do DOACs reduce intracranial bleeding compared to VKAs, but post hoc analyses of pivotal trials showed better outcomes following major bleeding under DOACs compared to VKAs [103, 104, 105, 106]. Notably, no specific reversal agents were available at that time.

The management strategy of patients who bleed on OAC should be based on clinical assessment of the severity of bleeding, dose and timing of last OAC intake and thromboembolic risk. Traditional coagulation assays and, if available, measurement of DOAC activity (anti‐factor Xa level for apixaban, edoxaban and rivaroxaban, dilute thrombin time or ecarin clotting time for dabigatran) and DOAC plasma levels are also useful to assess anticoagulant status and guide management. General work‐up includes assessment of renal and hepatic function, platelet count and co‐medication. Mild and major non‐life‐threatening bleeding can usually be managed with delaying or discontinuing the next OAC dose, in addition to supportive measures including haemostasis, fluid replacement and blood products substitution. In case of life‐threatening bleeding, or bleeding in a critical site (intracranial, intraspinal, intraocular, intra‐articular, pericardial, airway, hemothorax, retroperitoneal, intra‐abdominal, intramuscular with compartment syndrome), OAC reversal is generally recommended [80, 107], especially if last (D)OAC intake is recent (< 8–12 h) and coagulation assays indicate significant residual anticoagulation. Andexanet alfa, an inactive factor Xa analogue, non‐specifically binds factor Xa inhibitors resulting in reversal of apixaban, edoxaban, rivaroxaban as well as heparins [108, 109]. Idarucizumab is an antibody that specifically reverses dabigatran [110]. Alternatively, prothrombin complex concentrate (PCC; preferably four‐factor PCC) or activated PCC (aPCC; depending on availability and local experience) may be used to normalize coagulation parameters under any DOAC [111, 112, 113]. Fresh frozen plasma (FFP) is inadequate to reverse DOACs as very large volumes would have to be administered to compensate for the inhibition of newly administered coagulation factors by circulating DOACs. Activated factor VIIa is typically not recommended for DOAC reversal given the scarcity of data and substantial pro‐thrombotic effect [80, 107]. For dabigatran, haemodialysis may be used for rapid elimination if idarucizumab is not available. For patients on VKAs, reversal should be achieved preferably with PCCs [114] at a dose adjusted for INR and weight: 25 U/kg if INR 2–4, 35 U/kg if INR 4–6, 50 U/kg if INR > 6 (maximum dose 5000 U) [114, 115]. FFP can be used as an (inferior) alternative if PCCs are unavailable [114]. Vitamin K (up to 10 mg depending on INR) reduces INR within 4–6 h if administered intravenously and within 18–24 h if administered orally [116], which makes it unsuitable as a standalone reversal strategy in emergency situations.

Most patients derive net clinical benefit of OAC resumption after a major bleed, and DOACs appear preferable to VKAs [117]. The risk–benefit assessment of restarting OAC should take into account thromboembolic risk, bleeding location, reversible factors contributing to the bleed and whether the source of bleeding has been identified and treated. In patients with a CHA_2_DS_2_‐VA ≤ 1, OAC discontinuation may be considered if the risk of bleeding recurrence is deemed significant, whereas a CHA_2_DS_2_‐VA ≥ 4 generally identifies patients who would benefit from resumed OAC [107]. The timing of OAC reinitiation remains an area of significant uncertainty and should be decided in a multidisciplinary team. In most situations, parenteral anticoagulation may be resumed under close monitoring 1–3 days after haemostasis. When rebleeding risk is high, UFH is preferable given its short half‐life and antidote availability (protamine). Prophylactic‐dose parenteral anticoagulation may be used temporarily if bleeding risk is prohibitively high. Following intracranial haemorrhage, it is generally recommended to delay OAC reinitiation for ≥ 4 weeks [107, 118]. In specific situations, such as spontaneous intracerebral haemorrhage caused by amyloid angiopathy, or spontaneous subdural hematoma, rebleeding risk may be particularly high and extreme caution is advised if OAC resumption is considered. Modifiable bleeding risk factors should be reassessed and optimized as discussed above.

## 9 Anticoagulation in Specific Clinical Settings

### 9.1 Cardioversion of AF

Cardioversion of AF, whether pharmacological or electrical, is associated with a transient but substantial (> 10‐fold) increase in thromboembolic event rates within the 7 days that follow sinus rhythm restoration [119, 120, 121, 122]. The most likely mechanism is atrial stunning promoting de novo thrombus formation upon sinus rhythm restoration [122, 123, 124, 125]. While there is no randomized trial comparing OAC to no OAC in patients undergoing cardioversion, there is indisputable observational evidence that OAC reduces the risk of cardioversion‐related stroke by 60%–80% [119, 126, 127]. Importantly, the benefit of peri‐cardioversion OAC appears to be independent of chronic thromboembolic risk, and patients at low risk (e.g., CHA_2_DS_2_‐VA score of 0) also exhibit substantial benefit from OAC in the setting of cardioversion [127]. For these reasons, peri‐cardioversion OAC is recommended for all eligible patients undergoing cardioversion of AF regardless of CHA_2_DS_2_‐VA score, that is, including patients who do not have an indication to long‐term OAC. It might be safe to withhold OAC for patients without thromboembolic risk factors (e.g., CHA_2_DS_2_‐VA score of 0) undergoing cardioversion within < 12 h [128] of AF onset (assuming AF duration is clearly established, ideally documented objectively), but this remains an area of uncertainty and the default strategy should be OAC [11, 16]. Current recommendations are to prescribe OAC for ≥ 3 weeks before and ≥ 4 weeks after cardioversion [11, 16]. Alternatively, early cardioversion can be performed by ruling out intracardiac thrombus with imaging and introducing OAC immediately before cardioversion and for ≥ 4 weeks thereafter [129]. In case of clear AF duration < 24–48 h, early cardioversion without imaging (with OAC immediately before and ≥ 4 weeks after cardioversion) is generally considered safe [11, 16], even though there is scarce evidence. The safety of these recommendations has been validated in large prospective trials [129, 130, 131, 132], but the optimal duration of pre‐ and post‐cardioversion OAC remains unclear since alternative durations have not been tested in randomized trials [122]. Likewise, the benefit of imaging itself has not been formally established.

Meta‐analyses of randomized trials found DOACs to be at least non‐inferior to VKAs for the prevention of cardioversion‐related stroke [133, 134]. Given that DOACs exhibit a favourable safety profile, predictable pharmacokinetics, better ease of use and allow shorter time to cardioversion compared to VKAs [135], DOACs are recommended in preference to VKAs for peri‐cardioversion anticoagulation [11, 16]. When an early cardioversion strategy is pursued, DOACs should be administered per os ≥ 2 h (apixaban and edoxaban) to ≥ 4 h (rivaroxaban) before cardioversion [130, 131, 132]. For apixaban, a loading dose of 10 mg (5 mg if criteria for reduced dosing are met) should be administered as the starting dose; alternatively, cardioversion can be performed after five doses (2.5 days) without an initial loading dose [130]. Dabigatran was not specifically validated for early cardioversion, but pharmacokinetic data showed that maximum plasma concentration is attained after approximately 2 h. If anticoagulation by VKA is deemed preferable, early cardioversion may be performed after ≥ 1 dose of parenteral anticoagulant (e.g., enoxaparin 1 mg/kg s.c. twice daily), which should be continued until an INR ≥ 2.0 is achieved [130, 132].

### 9.2 Catheter Ablation

Catheter ablation of AF is the most effective rhythm control therapy and provides symptom relief [136], prevents disease progression [137] and improves prognosis in selected patients [138, 139, 140, 141]. Successful catheter ablation could also reduce long‐term stroke risk [66, 67, 68].

While the safety of AF ablation has improved over the years [142, 143, 144], the procedure remains associated with a non‐trivial risk of haemorrhagic and thromboembolic complications. In modern cohorts [144] and randomized trials [143], clinical stroke or transient ischaemic attack was reported in 0.12%–0.17% of procedures and pericardial effusion/tamponade in 0.36%–0.78%. Periprocedural clot formation and embolism may be due to catheter dwelling in the left atrium, ablation, or sinus rhythm restoration (atrial stunning); additionally, air embolism may result from catheter exchanges through the transseptal sheath [145].

The optimal balance between stroke prevention and periprocedural bleeding has been the subject of numerous studies. While historical practice was to discontinue VKAs with heparin bridging, observational studies [146] and one randomized trial [147] showed that uninterrupted VKA led to fewer thromboembolic complications without increasing the risk of major bleeding, and minor bleeding was in fact reduced. Regarding DOACs, a meta‐analysis of randomized trials found uninterrupted DOACs to reduce the risk of major bleeding compared to uninterrupted VKAs without significant difference in other outcomes [148]. Multiple randomized trials and observational studies have also compared uninterrupted DOACs to minimally interrupted DOACs, that is, skipping a single dose on the morning of the procedure without heparin bridging. Meta‐analyses of these studies found similar safety and efficacy compared to uninterrupted DOACs [149, 150, 151]. However, data from systematic brain MRI showed that the minimally interrupted strategy was associated with a higher risk of silent brain infarct [151]; the clinical significance of these lesions remains yet unclear.

To summarize, DOACs are preferable to VKAs, and uninterrupted OAC is superior to interrupted OAC with bridging. A strategy of minimally interrupted DOAC is a reasonable alternative to uninterrupted DOAC with comparable safety and efficacy [152]. In practice, DOACs are typically resumed 4 h after haemostasis. During the periprocedural period, once‐daily DOACs are preferably administered in the evening [153]. For patients taking a DOAC once daily in the morning, the intake schedule may be progressively shifted to the evening over 3 days (delaying the next dose by 4 h each day) in the weeks before the procedure.

The optimal duration of pre‐ and post‐ablation OAC is not well established. Current recommendations [152], based on expert opinion and extrapolated from rates of intra‐atrial thrombus in various clinical settings, are to administer OAC for ≥ 3 weeks before catheter ablation in most patients (with the possible exception of those with paroxysmal AF and a CHA_2_DS_2_‐VA score of 0). Imaging to rule out atrial thrombus is appropriate when patients with an indication to OAC have not received anticoagulation therapeutically for ≥ 3 weeks. Systematic imaging regardless of preprocedural anticoagulation is also deemed reasonable in patients at high risk of thrombus (CHA_2_DS_2_‐VASc score ≥ 3, persistent AF, hypertrophic cardiomyopathy, cardiac amyloidosis, or rheumatic heart disease) [152].

Based on expert consensus [152], OAC is recommended for ≥ 2 months after AF ablation, regardless of chronic thromboembolic risk, due to a transient prothrombotic state resulting from ablation‐related endothelial damage, inflammation and atrial stunning. Beyond this period, the default strategy is to continue long‐term OAC based on traditional thromboembolic risk estimation and regardless of the perceived success or failure of catheter ablation [11, 16]. Recently, however, two randomized trials, ALONE‐AF [67] and OCEAN [68], demonstrated the safety of OAC discontinuation ≥ 12 months after successful catheter ablation, defined as the absence of clinical evidence of atrial arrhythmia recurrence for ≥ 12 months based on at least 2 Holter monitoring sessions. OAC discontinuation resulted in a lower rate of bleeding events without a significant increase in systemic embolism. Of note, CHA_2_DS_2_‐VASc score was relatively low in both trials (median of 2 in ALONE‐AF and mean of 2.2 in OCEAN), but subgroup analysis found consistent results across CHA_2_DS_2_‐VASc categories. It may therefore be reasonable to discontinue OAC in selected patients who exhibit no recurrent atrial arrhythmia for ≥ 12 months after ablation. The safety of such a strategy is, however, not well established as practical questions remain to be clearly defined, including eligible patient profiles and post‐ablation rhythm monitoring. It should also be noted that long‐term follow‐up data > 3 years is currently lacking. To maximize safety, and given that post‐ablation recurrences are often asymptomatic [154], long‐term follow‐up with systematic rhythm monitoring appears reasonable and OAC should be resumed in case of arrhythmia recurrence [67, 152]. In ALONE‐AF, rhythm monitoring consisted of symptom‐driven assessments supplemented by 24–72 h Holter monitoring every 6 months. At 2 years after randomization, 9.2% of the patients exhibited atrial arrhythmia recurrence, prompting resumption of OAC. In the OCEAN trial, no rhythm monitoring was mandated by protocol during the study period.

### 9.3 Perioperative Management

**FIGURE 1:**
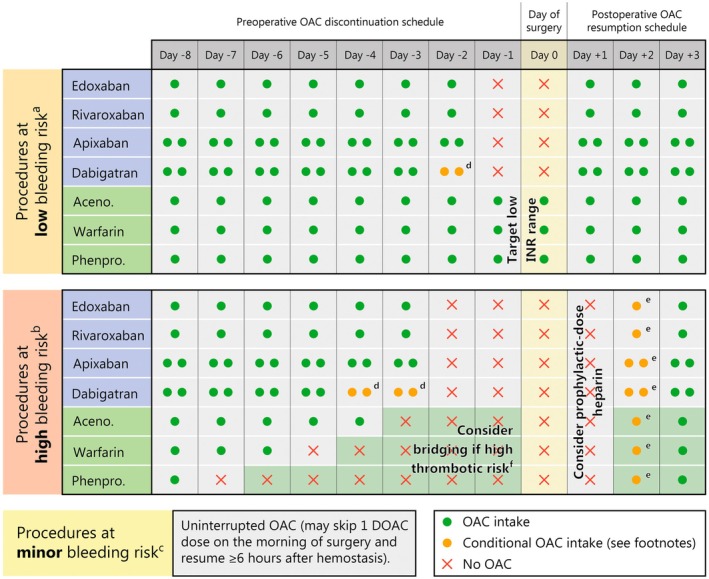
Perioperative OAC schedule according to procedure‐related bleeding risk and type of OAC. ^a^Procedures at low bleeding risk include: Abdominal surgery (cholecystectomy, hernia repair, colon resection), breast surgery, complex dental procedures (multiple tooth extractions), endoscopy with simple biopsy, gastroscopy or colonoscopy with simple biopsy, large‐bore needle procedures (e.g., bone marrow or lymph node biopsy), non‐cataract ophthalmic surgery, small orthopaedic surgery (foot, hand arthroscopy). ^b^Procedures at high bleeding risk include: Abdominal surgery with liver biopsy, extracorporeal shockwave lithotripsy, extensive cancer surgery (e.g., pancreas, liver), neuraxial (spinal or epidural) anaesthesia, neurosurgery (intracranial, spinal), major orthopaedic surgery, procedures with vascular organ biopsy (kidney or prostate), reconstructive plastic surgery, specific interventions (colon polypectomy, lumbar puncture, endovascular aneurysm repair), thoracic surgery, lung resection surgery, urological surgery (prostatectomy, bladder tumour resection), vascular surgery (e.g., aortic surgery, vascular bypass). ^c^Procedures at minor bleeding risk include: Cardiac device implantation, most percutaneous catheter ablation procedures, most percutaneous angioplasty procedures, cataract or glaucoma procedures, minor dental procedures (extractions [1–3 teeth], periodontal surgery, implant positioning, endodontic [root canal] procedures, subgingival scaling/cleaning), endoscopy without biopsy or resection, superficial surgery (e.g., abscess incision, small skin excisions/biopsy). ^d^Only administer if creatinine clearance ≥ 50 mL/min, otherwise withhold dabigatran. ^e^Only resume OAC 48 h after high‐risk surgery if haemostasis has been secured, otherwise withhold until 72 h after surgery. ^f^Patients at high thrombotic risk for whom bridging (days shaded in green) should be considered include: Patients with mitral or tricuspid mechanical valve prostheses, patients with older‐generation aortic mechanical valve prostheses, patients with current‐generation aortic mechanical valve prostheses and additional risk factors (AF, previous thromboembolism, severe left ventricular dysfunction, prothrombotic state), AF patients with a CHA_2_DS_2_‐VASc score > 5–6, patients with recent embolic stroke < 3 months, intracardiac thrombus, or thrombophilia. Aceno., acenocoumarol; AF, atrial fibrillation; DOAC, direct oral anticoagulant; OAC, oral anticoagulant; phenpro., phenprocoumon.

For elective procedures, a standardized approach to the perioperative management of patients on OAC consists in classifying procedure‐related bleeding risk as minimal, low, or high, based on bleeding risk, ease of controlling bleeding and risk of adverse outcome if bleeding occurs [80, 115, 155]. Bleeding risk categories of different types of procedures and the corresponding perioperative OAC management are summarized in Figure 1. For procedures at minimal bleeding risk, DOACs may be continued uninterrupted or minimally interrupted (skipping one dose on the morning of the procedure and resuming ≥ 6 h after haemostasis). For procedures at low bleeding risk, DOACs should typically be withheld 1 day before the procedure (i.e., last dose is administered on Day −2) and resumed 1 day after. For procedures at high bleeding risk, DOACS should generally be withheld 2 days before (i.e., last dose is administered on Day −3) and resumed 2–3 days after the procedure (heparin at prophylactic dose should be considered between surgery and resumption of DOAC). For patients with renal dysfunction, dabigatran should be discontinued for longer due to significant renal elimination [115].

For patients on VKAs, procedures with minimal or low bleeding risk can generally be performed on uninterrupted VKA, with INR monitoring and targeting the lower portion of the therapeutic range. For procedures at high bleeding risk, VKAs should be interrupted to achieve an INR ≤ 1.5 on the day of the procedure. Acenocoumarol, warfarin and phenprocoumon should be discontinued 3, 5 and 7 days before surgery, respectively (i.e., last dose is administered on day −4, −6 and −8, respectively) [115]. Randomized trials comparing heparin bridging to no bridging in AF patients found bridging to increase bleeding risk without reducing thromboembolic events [156, 157]. In patients with mechanical heart valves, both observational and randomized data showed no significant benefit of bridging in this population [156, 158, 159]. As a result, current guidelines only recommend to consider bridging in selected patients at particularly high thromboembolic risk, including AF patients with a CHA_2_DS_2_‐VASc score > 5–6, patients with mitral or tricuspid mechanical valve prostheses, patients with older‐generation aortic mechanical valve prostheses, patients with current‐generation aortic mechanical valve prostheses and additional risk factors (AF, previous thromboembolism, severe left ventricular dysfunction, prothrombotic state), patients with recent embolic stroke < 3 months, intracardiac thrombus, or thrombophilia [115]. Low‐molecular‐weight heparin (LMWH) and unfractionated heparin (UFH) have shown similar efficacy and safety for VKA bridging [160], but LMWH is used more commonly due to its ease of use and more predictable dose–response pattern [159]. LMWH/UFH is typically started as soon as INR drops below the inferior limit of therapeutic range. LMWH should be discontinued > 12 h before surgery (e.g., last dose 24 h before surgery) and UFH should be discontinued 6 h before surgery. LMWH/UFH should be resumed 48–72 h after high‐risk surgery depending on bleeding and thrombotic risk.

For patients on DOACs requiring urgent surgery, the management strategy should take into account the degree of urgency of the procedure and the timing of last DOAC intake. Coagulation tests, DOAC‐specific assays and DOAC plasma levels are useful to guide pre‐ and post‐operative treatment. While there are no evidence‐based cut‐offs, some authors have proposed empirically that an anti‐factor Xa level < 50 ng/mL or dilute thrombin time < 50 s may allow for surgery to be done safely without DOAC reversal [155]. If deemed safe, deferral of surgery to > 12 h after last DOAC intake (> 24 h if eGFR < 50 mL/min) is the preferred strategy [108]. If immediate surgery is warranted and last DOAC intake is < 12 h, DOAC reversal may be advisable depending on bleeding risk (reversal agents discussed in Section 8). For patients on VKAs requiring urgent surgery with high bleeding risk warranting an INR ≤ 1.5, PCCs are the preferred reversal agent [114], while intravenous vitamin K may be sufficient if surgery can be safely deferred for at least 4–6 h [116].

### 9.4 Elderly and Frail Patients

The prevalence of AF increases exponentially with age, from < 1% among adults < 60 years to 4%–6% of primary care patients aged 65–69 years and 17%–29% of those aged ≥ 85 years [161]. Advancing age is associated with greater risks of both bleeding and stroke [59, 70]. Additionally, frailty, defined as reduced physiologic reserve with increased vulnerability to stressors, is present in 40%–50% of older adults with AF [162, 163, 164]. Frailty itself is also associated with worse outcomes including stroke, bleeding and death [162]. Likewise, older adults with AF exhibit a high burden of falls, dependency for daily activities, polypharmacy and multimorbidity [164]. In a retrospective cohort study of 433,123 patients with incident AF and a mean age of 80 years, mortality within 12 months of a new AF diagnosis was 25%, highlighting the fragility of this population [165].

As a result, age might affect the risk–benefit profile and net benefit of OAC. The elderly population is highly heterogeneous and underrepresented in randomized trials, making evidence‐based recommendations limited. Nevertheless, post hoc analyses of randomized trials and observational data provide compelling evidence that the prognostic benefit of OAC for reducing thromboembolism and all‐cause mortality is preserved in elderly patients, including the very elderly (e.g., ≥ 90 years) [70, 166, 167, 168]. Repetitive falls often elicit concern for bleeding risk, but even when frequent, falls do not negate the benefit of OAC [169] and should not lead to withdrawal of therapy. OAC should therefore be the default strategy in all eligible elderly patients with AF. Of note, other authors have argued for a more nuanced approach, based in part on the potential futility of continued OAC in patients with very high competing risk of death unrelated to AF (e.g., end‐stage disease, < 6–12 months life expectancy) [164, 170]. For example, some observational data suggests that the mortality benefit of OAC may vanish in patients aged ≥ 85 years with moderate to severe dementia compared to those without dementia, while the benefit of reduced thromboembolic events is preserved [171]. In our opinion, more data is needed to withhold OAC in patients without established contraindications.

Similarly to the general AF population, DOACs are preferable to VKAs in elderly patients [70, 71]. Given their superior risk–benefit profile, the net clinical benefit of OAC is maintained at more advanced ages with DOACs compared to VKAs [170]. Post hoc analyses of randomized trials found significant effect modification by age for bleeding risk with dabigatran and rivaroxaban, while safety was unaffected by age for apixaban and edoxaban [70]. Similar findings were reported in a large registry study [167]. Ongoing trials with head‐to‐head comparisons of different DOACs may help clarify these issues.

### 9.5 Pregnancy

Pregnant patients with AF are best managed in a multidisciplinary team, ideally involving cardiologists with experience in maternal medicine [11, 172]. Thromboembolic risk may be assessed with the CHA_2_DS_2_‐VA score and indications to OAC are the same as in non‐pregnant patients [11]. DOACs are not recommended during pregnancy because of safety concerns. VKAs offer the best protection against thromboembolism (especially in patients with mitral stenosis or mechanical heart valves), but are associated with embryopathy/foetopathy in a dose‐dependent fashion and particularly during the first trimester [173]. Additionally, because foetal anticoagulation may remain for 8–10 days after VKA discontinuation, vaginal delivery should be avoided within < 2 weeks of last VKA intake to prevent foetal intracranial haemorrhage. As a result, therapeutic anticoagulation with LMWH is typically recommended during the first trimester, unless therapeutic INR is achieved with low VKA doses (daily dose ≤ 5 mg for warfarin, ≤ 3 mg for phenprocoumon, ≤ 2 mg for acenocoumarol), in which case VKA may be continued throughout the first trimester [172]. Starting from week 13, and until the 36th week, VKAs are generally preferred for women at high thrombotic risk (AF with significant mitral stenosis, mechanical heart valves). Planned delivery is recommended, with a switch to LMWH or UFH at the 36th week or 2 weeks before planned delivery. For women at high thrombotic risk, bridging with UFH ≥ 36 h before delivery and UFH discontinuation 4–6 h before delivery is recommended. Post‐partum resumption of anticoagulation with LMWH or UFH should be decided jointly with obstetric, anaesthetic, haematology and cardiology teams. VKA resumption should be delayed to at least 1–2 weeks post‐partum due to the risk of late obstetric bleeding [174, 175]. DOACs are preferably avoided during breastfeeding due to a lack of safety data. Pharmacological data indicates very low drug concentrations in neonates of women taking dabigatran or rivaroxaban [176, 177], which may be used cautiously if necessary [172].

## 10 Areas of Uncertainty and Future Perspectives

Table 4 summarizes key areas of uncertainty regarding OAC in AF patients, along with currently available evidence and perspectives for future research and further improvements in patient outcomes.

**TABLE 4:** Key areas of uncertainty in anticoagulation of AF patients and future perspectives.

Area of uncertainty	Current evidence	Future perspectives
*Thromboembolic risk assessment* remains relatively imprecise and heterogeneous groups may be stratified together.	Established risk scores (e.g., CHA_2_DS_2_‐VA(Sc)) exhibit variable accuracy across populations [28]. CHA_2_DS_2_‐VA(Sc) score shown to be inadequate to determine OAC indication in specific populations, including hypertrophic cardiomyopathy [29], cardiac amyloidosis [30] and rheumatic heart disease [31]	Other, not yet clearly identified populations may be misclassified by traditional risk scores. More individualized risk estimation tools might be useful (circulating biomarkers, imaging?)
In *device‐detected AF* lasting 6 min to 24 h, thromboembolic risk assessment and threshold for OAC are not clearly established	In CHA_2_DS_2_‐VASc ≥ 2–3, OAC yields 3 fewer ischemic strokes per 1000 patient‐years at the cost of 7 more major bleeding events per 1000 patient‐years [46]. Net benefit of OAC may be greater in CHA_2_DS_2_‐VASc ≥ 4–5, higher device‐detected AF burden, vascular disease [42, 47, 48]. Device‐detected AF associated with high risk of disease progression [49]	Clearer definition of the threshold for net clinical benefit of OAC in device‐detected AF (higher CHA_2_DS_2_‐VA than in clinical AF?) might improve management and outcomes
In *trigger‐induced AF*, the benefit of long‐term OAC and optimal timing of OAC introduction are not clearly established	New‐onset AF with an acute precipitating factor associated with ~40% risk of AF recurrence at 5 years [50]. Risk of long‐term AF recurrence after acute medical illness > non‐cardiac surgery > cardiac surgery [50]. Observational data suggests potential benefit of long‐term OAC in these populations [51, 54, 55, 56]. Introduction of OAC in the acute phase of sepsis may increase bleeding without reducing stroke [57]	Ongoing randomized trials (e.g., PACES [NCT04045665] and ASPIRE‐AF [NCT03968393]) may clarify management. More individualized tools to estimate AF recurrence risk might be useful
The safety of *OAC discontinuation after successful catheter ablation* of AF is not well established	A meta‐analysis of randomized trials suggested that catheter ablation reduces long‐term stroke risk [66]. Randomized trials have shown that OAC could be safely discontinued after catheter ablation without arrhythmia recurrence for ≥ 1 year in selected patients [67, 68]	Post‐ablation rhythm monitoring necessary for safe OAC discontinuation remains to be clearly defined. The population that may safely benefit from post‐ablation OAC discontinuation remains to be clearly defined (e.g., CHA_2_DS_2_‐VA threshold? Estimated risk of AF recurrence?). Long‐term follow‐up data (> 3 years) is needed
The role of *LAA occlusion versus OAC* is not established	Randomized trials have shown non‐inferior protection against thromboembolism compared to OAC in selected AF patients [65], but complications specific to LAA occlusion need to be weighed against OAC bleeding risk	Newer and upcoming LAA occlusion devices and techniques may further improve safety and efficacy. The specific patient characteristics and settings in which LAA occlusion might provide net clinical benefit over long‐term OAC remain to be defined
The *comparative safety and efficacy of different DOACs* is not established	Apixaban, dabigatran, edoxaban and rivaroxaban have different pharmacologic properties, posology and exhibit differences in safety and efficacy profiles compared to VKAs. Apixaban was safer than rivaroxaban in a randomized trial on acute venous thromboembolism [178]. No randomized trial comparing different DOACs head‐to‐head in AF is currently available	Ongoing randomized trials (e.g., VALIANT‐AF‐T, NCT06953726) may clarify if specific DOACs are safer and/or more effective for long‐term OAC
The net clinical benefit and optimal posology of *OAC in CKD stages 4 and 5* (including dialysis) is not well established	Available data suggests that DOACs may be safer and at least as effective as VKAs [99]	The optimal molecule and dose remain to be determined and will likely condition the net clinical benefit of OAC in this population
The optimal *duration of pre‐ and post‐cardioversion OAC* is not established	Large prospective trials have validated the safety of currently recommended protocols (OAC ≥ 3 weeks pre‐ and ≥ 4 weeks post‐cardioversion, or imaging with OAC immediately before and ≥ 4 weeks post‐cardioversion) [129, 130, 131, 132], but alternative OAC durations have not been tested in randomized trials [122]. The utility of pre‐cardioversion imaging has not been formally established	If shown to be safe, shorter pre‐cardioversion delays may improve rhythm outcomes

Abbreviations: AF denotes atrial fibrillation; CKD, chronic kidney disease; DOAC, direct oral anticoagulant; LAA, left atrial appendage; OAC, oral anticoagulation; VKA, vitamin K antagonist.

## 11 Conclusion

Practical recommendations for OAC in AF patients are summarized in the graphical abstract. Key practical takeaways, including thresholds and definitions critical to decision‐making, are summarized in Table 5. OAC is the mainstay of stroke prevention in AF and provides net clinical benefit in the vast majority of patients whose stroke risk exceeds 1%–2%/year. With few specific exceptions, DOACs are preferable to VKAs, including in patients with prior bleeding, frail elderly patients, and those with CKD. Modifiable bleeding risk factors should be assessed and mitigated. High bleeding risk per se does not represent a contraindication to OAC. Absolute contraindications are very rare and most are temporary. Dose adaptations of DOACs to renal function, age and body weight should follow strict evidence‐based criteria to avoid inappropriate underdosing. Follow‐up should be systematic and involve reassessment of adherence, thrombotic and bleeding risk, co‐medication and adequacy of the dosing regimen. Nurse‐led AF clinics help coordinate follow‐up and improve outcomes. It is currently recommended to pursue OAC as a lifelong therapy, but recent data suggested that discontinuing OAC after successful catheter ablation of AF and/or left atrial appendage closure could be safe. Notably, therapies to prevent stroke and systemic embolism should be implemented as part of a structured and integrated approach to AF management, which should also include the management of comorbidities and AF risk factors, symptom mitigation by rate and rhythm control, and dynamic reassessment (e.g., AF‐CARE framework).

**TABLE 5:** Key practical takeaways on the management of OAC in AF patients.

Clinical decision/setting	Recommendation
Threshold for net clinical benefit of OAC	Thromboembolic risk ≥ 1%/year (stronger recommendation if ≥ 2%/year), corresponding to a CHA_2_DS_2_‐VA ≥ 1 (stronger recommendation if ≥ 2)
Indication to OAC in device‐detected AF	< 6 min: no indication to OAC Between 6 min and 24 h: area of uncertainty, likely benefit of OAC in some patients (see Table 4). Follow‐up important (disease progression). >24 h: OAC generally recommended as in clinical AF
Indication to OAC in trigger‐induced AF (post‐operative or acute medical illness)	Area of uncertainty: Consider OAC initiation as soon as deemed safe from a surgical standpoint Reassess indication to long‐term OAC at 60 days depending on rhythm status ○ Consider long‐term OAC, especially if trigger was non‐cardiac surgery or acute medical illness ○ Consider OAC discontinuation if trigger was hyperthyroidism and normal thyroid function has been restored along with sinus rhythm
Patients in whom VKAs are preferred to DOACs	Mechanical heart valves Significant mitral stenosis (≥ moderate) Antiphospholipid syndrome Child‐Turcotte‐Pugh C cirrhosis
Structured follow‐up of patients on OAC	As part of an integrated patient‐centred approach to AF management (e.g., AF‐CARE): Reassess thromboembolic risk ○ Inquire about thromboembolic events ○ Review and optimize thromboembolic risk factors Reassess bleeding risk ○ Inquire about bleeding events ○ Review and optimize modifiable bleeding risk factors Inquire about side‐effects Review and optimize adherence (+educate on dosing, intake schedule, adherence aids, dealing with lapses in dosing, involve family members when appropriate) Review co‐medication for drug interactions and bleeding risk ≥ 1×/year blood sample to follow haemoglobin, renal and liver function Reassess adequacy of dosing based on evidence‐based criteria
Minimal duration of peri‐cardioversion OAC	Optimal duration unknown, current recommendations validated in noncomparative studies: Pre‐cardioversion: ○ ≥ 3 weeks or ○ immediately before cardioversion (2–4 h depending on choice of OAC) + imaging to rule out intracardiac thrombus • Post‐cardioversion: ≥ 4 weeks (long term if CHA_2_DS_2_‐VA ≥ 1)
Minimal duration of periprocedural OAC for catheter ablation of AF	Optimal duration unknown, current recommendations validated in noncomparative studies: Pre‐ablation: ○ ≥ 3 weeks (+imaging in high‐risk patients) or ○ imaging to rule out intracardiac thrombus Post‐ablation: ≥ 2 months (long term if CHA_2_DS_2_‐VA ≥ 1, discontinuation might be safe in specific settings)
OAC resumption after major bleeding	Area of uncertainty: Multidisciplinary decision with the team involved in the management of bleeding; consider thromboembolic risk, bleeding location, reversible bleeding risk factors and reversibility of the source of bleeding ○ CHA_2_DS_2_‐VA ≤ 1: consider OAC discontinuation if significant rebleeding risk ○ CHA_2_DS_2_‐VA ≥ 4: OAC resumption usually beneficial
Timing of OAC resumption after major bleeding	Multidisciplinary decision with the team involved in the management of bleeding; ○ Typically 1–3 days after haemostasis, preferably with parenteral anticoagulation ○ ≥ 4 weeks after intracranial haemorrhage (area of uncertainty)
OAC in elderly and frail patients	Prognostic benefit of OAC generally preserved DOACs generally preferable to VKAs Repetitive falls should not lead to withdrawal of therapy

Abbreviations: AF, atrial fibrillation; DOAC, direct oral anticoagulant; OAC, oral anticoagulant; VKA, vitamin K antagonist.

## Funding

The authors have nothing to report.

## Conflicts of Interest

N.J. received a scholarship from the Swiss National Science Foundation (Grant No. 225328).

## Data Availability

Data sharing is not applicable to this article as no new data were created or analysed in this review.
